# Round the Hospitals

**Published:** 1916-09-09

**Authors:** 


### ROUND THE HOSPITALS.

Miss Mary Girdlestone, who for nearly twenty years has been matron at Crumpsall Infirmary, Manchester, has resigned her post, and will relinquish her work next month. Since her appointment in January 1897 she has effected wonders at Crumpsall, and the high quality of the work done by her and her staff has deservedly won for itself wide recognition from those whose duty and privilege it is to appraise the value of superintendence and nurse training in our voluntary hospitals and Poor-Law infirmaries. Miss Girdlestone's work was specially referred to in the article on the Crumpsall Workhouse Infirmary in The Hospital of December 4, 1909 ("Reports on Hospitals of the United Kingdom," by Sir Henry Burdett), and our readers will do well to turn to that article and the introduction on page 273 to appreciate what it is possible to achieve in the administration and nursing arrangements of a Poor-Law institution. Miss Girdlestone, who was trained at St. Bartholomew's Hospital, was subsequently superintendent of nursing at Hampstead Workhouse Infirmary, a post she resigned on her appointment to Crumpsall. The good wishes and affection of the many Crumpsall trained nurses, and of the nursing profession as a whole, will go with Miss Girdlestone on her retirement.

The award of the Military Medal to six ladies, five of whom are trained nurses, announced in a Supplement to the *London Gazette* of September 1, is the first occasion on which this medal has been granted to women. Their inclusion was effected by Royal Warrant dated June 21, which provided that the Military Medal may, in exceptional circumstances, on the special recommendation of a Commander-in-Chief in the field, be awarded either to British women or to foreign persons who have shown bravery and devotion under fire. Four of the nurses now honoured were reported as wounded in the casualty lists of August 17. Their names are Matron M. M. Tunley, R.R.C., Q.A.I.M.N.S.; Sisters Beatrice A. Allsop and Norah Easeby, Q.A.I.M.N.S.R.; and Staff Nurse Jean S. Whyte, T.F.N.S. Staff Nurse Ethel Hutchinson, Q.A.I.M.N.S.R., has also been awarded the Military Medal. Matron Tunley was trained at Leeds General Infirmary, Miss Whyte at the Western Infirmary, Glasgow, Miss Allsop and Miss Easeby at St. Thomas's Hospital, and Miss Hutchinson at the London Hospital. All have been serving in France, and Matron Tunley has been twice mentioned in despatches and has received the Royal Red Cross, first class. We congratulate these nurses on the honour conferred upon them. The nursing profession has good reason to be proud of this, the first occasion of the award of this medal to nurses.

After the war many things must change for the better. The war has proved a great leveller and has brought all classes into closer intercommunion and co-operation, with results which are producing mutual respect and a better understanding of various points of view. Matrons and lady superintendents of training schools and hospitals generally, if they keep in touch with present movements, should have begun to realise that probationers and nurses should be taught the importance of accepting the patient as a guest and of maintaining towards him or her the recognition that they have feelings and points of view which should be respected. In the best schools familiarity between the staff and the patient is properly discouraged. Some of the fruits of nurses' free and easy language we frequently have brought to our notice through the complaints from superintendents and private and district nurses that probationers and others fall into the habit of calling their patients "Daddy" and "Granny" and so forth. There is nothing which a self-respecting patient resents more at heart than this vulgar habit.

That this is so is shown by experience in the nursing of the sick in their own homes. There the hospital-trained nurse in private work or work in the district who continues the habit referred to and often adds "dear" when addressing a patient properly causes much resentment by both patients and their friends. The patients in the hospitals are under a sense of indebtedness for the free treatment they receive, and their natural delicacy causes them to remain silent, however much they may dislike the familiarity. When the patient is at home he is independent, and his resentment finds expression not by direct complaint but by telling the nurse she need not call again. That this habit has grown to an abuse is indicated by the case of a clergyman who, when a patient in hospital, was addressed sometimes as "Daddy" and sometimes as "Dear." We were inclined to attach small importance to this matter at the outset, but as the complaints increase and the evidence accumulates it seems desirable to call attention to this regrettable habit. We shall be glad to have for publication the experiences of some superintendents, district nurses, and others who in the course of their work have received complaints from patients and realised their resentment.

The trustees of the Lady Priestley Lectureship have reprinted the exhaustive address of the Duchess of Marlborough entitled "Saving the Children." This address is practical and interesting, and many of our readers may like to know that it is published by the National Health Society, 53 Berners Street, price 1s., from whom copies can be obtained. This address is being sold to aid the funds of the Lady Priestley Lectureship, which are rather low at the present time, and might be added to with considerable benefit to the nursing profession and the public at large. Five guineas or more from those of us who can afford it, and smaller sums from others, would be well spent by intelligent philanthropists and busy workers who know and value the importance of hygiene and sanitation in all its aspects, not only to the children but to their elders of all ages and to the nation and Empire.

For Matrons' and Sisters' Appointments see p. vi.
For National Poor-law Officers' Association see pp. 547–8.

